# Supplementary material for: HIVprotI: an integrated web based platform for prediction and design of HIV proteins inhibitors
Source: J Cheminform. 2018 Mar 9;10:12. doi: 10.1186/s13321-018-0266-y (PMC5845081; doi:10.1186/s13321-018-0266-y)
Supplement: Supplementary file 2 — Additional file 2. Source code of HIVProtI web server. [file 13321_2018_266_MOESM2_ESM.zip › HIVprotI_Source-code/hivprotI/weka-3-6-6/doc/weka/core/Debug.Log.html]

Debug.Log


---


|  |  |  |
| --- | --- | --- |
| **Overview** \| **Package** \| **Class** \| **Tree** \| **Deprecated** \| **Index** \| **Help** \| **Weka's home** | |  |
| **PREV CLASS**   **NEXT CLASS** | **FRAMES**    **NO FRAMES**     **All Classes** |  |
| SUMMARY: NESTED \| FIELD \| CONSTR \| METHOD | DETAIL: FIELD \| CONSTR \| METHOD |  |


---


## weka.core Class Debug.Log

```
java.lang.Object
  weka.core.Debug.Log
```

**All Implemented Interfaces:**: java.io.Serializable, RevisionHandler

**Enclosing class:**: Debug

---

``` public static class Debug.Log extends java.lang.Object implements java.io.Serializable, RevisionHandler ```

A helper class for logging stuff. Uses the java.util.logging
package. If this approach seems an "overkill" (it can create quite a few
log files if used in different threads), one can use the
Debug.SimpleLog class.

**Version:**
:   $Revision: 7519 $

**Author:**
:   FracPete (fracpete at waikato dot ac dot nz)

**See Also:**: `Debug.SimpleLog`, Serialized Form

---

**Constructor Summary**

`Debug.Log()`
  
          default constructor, uses only stdout

`Debug.Log(java.lang.String filename)`
  
          creates a logger that logs into the specified file, if null then only
stdout is used.

`Debug.Log(java.lang.String filename,
int size,
int numFiles)`
  
          creates a logger that logs into the specified file, if null then only
stdout is used.


| **Method Summary** | |
| --- | --- |
| `java.lang.String` | `getFilename()`             returns the filename of the log, can be null |
| `int` | `getNumFiles()`             returns the number of files being used |
| `java.lang.String` | `getRevision()`             Returns the revision string. |
| `int` | `getSize()`             returns the size of the files |
| `void` | `log(java.util.logging.Level level, java.lang.String message)`             logs the given message |
| `void` | `log(java.util.logging.Level level, java.lang.String sourceclass, java.lang.String message)`             prints the given message with the specified level |
| `void` | `log(java.util.logging.Level level, java.lang.String sourceclass, java.lang.String sourcemethod, java.lang.String message)`             prints the given message with the specified level |
| `void` | `logSystemInfo()`             a convenience method for dumping the current system info in the log file |
| `static java.util.logging.Level` | `stringToLevel(java.lang.String level)`             turns the string representing a level, e.g., "FINE" or "ALL" into the corresponding level (case-insensitive). |
| `java.lang.String` | `toString()`             returns a string representation of the logger |

**Methods inherited from class java.lang.Object**

`equals, getClass, hashCode, notify, notifyAll, wait, wait, wait`

**Constructor Detail**

### Debug.Log

```
public Debug.Log()
```

:   default constructor, uses only stdout

---


### Debug.Log

```
public Debug.Log(java.lang.String filename)
```

:   creates a logger that logs into the specified file, if null then only
    stdout is used. It uses 1,000,000 bytes for file size and 1 file.

    **Parameters:**: `filename` - the file to log into

---


### Debug.Log

```
public Debug.Log(java.lang.String filename,
                 int size,
                 int numFiles)
```

:   creates a logger that logs into the specified file, if null then only
    stdout is used.

    **Parameters:**: `filename` - the file to log into: `size` - the size of the files in bytes: `numFiles` - the number of files for rotating


**Method Detail**

### stringToLevel

```
public static java.util.logging.Level stringToLevel(java.lang.String level)
```

:   turns the string representing a level, e.g., "FINE" or "ALL" into
    the corresponding level (case-insensitive). The default is ALL.

    :   **Parameters:**: `level` - the string to return a level for **Returns:**: the corresponding level or the default

---


### getFilename

```
public java.lang.String getFilename()
```

:   returns the filename of the log, can be null

    :   **Returns:**: the filename of the log

---


### getSize

```
public int getSize()
```

:   returns the size of the files

    :   **Returns:**: the size of a file

---


### getNumFiles

```
public int getNumFiles()
```

:   returns the number of files being used

    :   **Returns:**: the number of files

---


### log

```
public void log(java.util.logging.Level level,
                java.lang.String message)
```

:   logs the given message

    :   **Parameters:**: `level` - the level of severity: `message` - the message to log

---


### log

```
public void log(java.util.logging.Level level,
                java.lang.String sourceclass,
                java.lang.String message)
```

:   prints the given message with the specified level

    :   **Parameters:**: `level` - the level of logging: `sourceclass` - the class that logs the message: `message` - the message to print

---


### log

```
public void log(java.util.logging.Level level,
                java.lang.String sourceclass,
                java.lang.String sourcemethod,
                java.lang.String message)
```

:   prints the given message with the specified level

    :   **Parameters:**: `level` - the level of logging: `sourceclass` - the class that logs the message: `sourcemethod` - the method that logs the message: `message` - the message to print

---


### logSystemInfo

```
public void logSystemInfo()
```

:   a convenience method for dumping the current system info in the
    log file

    :   **See Also:**: `SystemInfo`

---


### toString

```
public java.lang.String toString()
```

:   returns a string representation of the logger

    :   **Overrides:**: `toString` in class `java.lang.Object`
    :   **Returns:**: a string representation of the logger

---


### getRevision

```
public java.lang.String getRevision()
```

:   Returns the revision string.

    :   **Specified by:**: `getRevision` in interface `RevisionHandler`
    :   **Returns:**: the revision


---


|  |  |  |
| --- | --- | --- |
| **Overview** \| **Package** \| **Class** \| **Tree** \| **Deprecated** \| **Index** \| **Help** \| **Weka's home** | |  |
| **PREV CLASS**   **NEXT CLASS** | **FRAMES**    **NO FRAMES**     **All Classes** |  |
| SUMMARY: NESTED \| FIELD \| CONSTR \| METHOD | DETAIL: FIELD \| CONSTR \| METHOD |  |


---
